# Eight-Year Follow-Up of Neuropsychiatric Symptoms and Brain Structural Changes in Fabry Disease

**DOI:** 10.1371/journal.pone.0137603

**Published:** 2015-09-04

**Authors:** Irene M. Lelieveld, Anna Böttcher, Julia B. Hennermann, Michael Beck, Andreas Fellgiebel

**Affiliations:** 1 Department of Psychiatry and Psychotherapy, University Medical Center of the Johannes Gutenberg-University, Mainz, Germany; 2 Villa Metabolica, Center for Pediatric and Adolescent Medicine, University Medical Center of the Johannes Gutenberg-University, Mainz, Germany; 3 Institute of Human Genetics, University Medical Center of the Johannes Gutenberg-University, Mainz, Germany; University of California, San Francisco, UNITED STATES

## Abstract

Brain structural alterations and neuropsychiatric symptoms have been described repeatedly in Fabry disease, yet cognitive deficits have been shown to be only mild. Here, we aimed to investigate neuropsychiatric symptoms and brain structure longitudinally. We expected no clinically relevant increase of neuropsychiatric symptoms in parallel to increased brain structural alterations. We assessed 14 Fabry patients (46.1 ± 10.8 years) who had participated in our investigation eight years ago. Patients engaged in neuropsychiatric testing, as well as structural magnetic resonance imaging and angiography to determine white matter lesions, hippocampal volume, and the diameter of the larger intracranial arteries. While Fabry patients did not differ on cognitive performance, they showed progressive and significant hippocampal volume loss over the 8-year observation period. White matter lesions were associated with older age and higher white matter lesion load at baseline, but did not reach statistical significance when comparing baseline to follow-up. Likewise, intracranial artery diameters did not increase significantly. None of the imaging parameters were associated with the neuropsychiatric parameters. Depression frequency reduced from 50% at baseline to 21% at follow-up, but it did not reach significance. This investigation demonstrates clinical stability in cognitive function, while pronounced hippocampal atrophy is apparent throughout the 8 years. Our middle-aged Fabry patients appeared to compensate successfully for progressive hippocampal volume loss. The hippocampal volume decline indicates brain regional neuronal involvement in Fabry disease.

## Background

Fabry disease (FD) is a rare hereditary x-linked lysosomal storage disorder that results from a deficient activity of the enzyme α-galactosidase A. Consequent lipid accumulation results in multiorgan pathology that predominantly affects tissues of cardiac or renal systems, and the central nervous system (CNS) [1]. CNS involvement precipitates cerebral micro- and macro angiopathy leading to stroke at an early age in FD with an estimated prevalence of 6.9% in men and 4.3% in female FD patients [1, 2]. CNS alterations reported include increased occurrence of white matter lesions (WML), dilation and tortuosity of the larger intracranial arteries i.e. [3, 4], signal enhancement of the lateral pulvinar on T1-weighted images, and hippocampal atrophy [5]. In non-Fabry cohorts WMLs have frequently been related to processing speed, memory, and executive functioning deficits [6]. Additionally, hippocampal volume (HV) decline can cause severe memory deficits and is a well-known predictor for Alzheimer’s disease, a disease known for its pronounced cognitive decline [7]. In FD cognitive deficits have been described primarily in attention, executive functions, and psychomotor performance [8, 9]. However, studies are inconclusive on the degree of these deficits and whether its development is a result of depressive symptoms or of neurological alterations. In non-FD subjects depression might be associated with increased WML load in older subjects [10], but Schermuly and colleagues (2011) could not find this relationship in FD patients [9]. In the same investigation they found that 60% of the 25 FD patients enrolled showed clinical depression compared to healthy controls. In fact, depression is by far the most frequently reported psychiatric complication of FD ranging from a 15% to 62.5% prevalence and can significantly affect disease burden. Nonetheless, it is unclear whether depression is a symptom occurring from FD specific CNS manifestations, or a syndrome arising due to an incurable painful disease.

Existing studies have only focused on neuropsychiatric and neurological FD symptoms cross-sectionally. However, longitudinal designs are necessary to determine the relationship between neuropsychiatric and neurological symptoms in FD. In line with our baseline investigation where FD patients and healthy controls only differed slightly in their cognitive performance [9], we intend to demonstrate that clinically relevant cognitive performance decline is also not indicated after eight years. Furthermore, we expect marked increases of WML-load and significant hippocampal atrophy longitudinally. In an exploratory analysis we investigate depressive symptom development, as well as changes in diameter of the larger intracranial arteries over time.

## Material and Methods

### Patients

**Fig 1:**
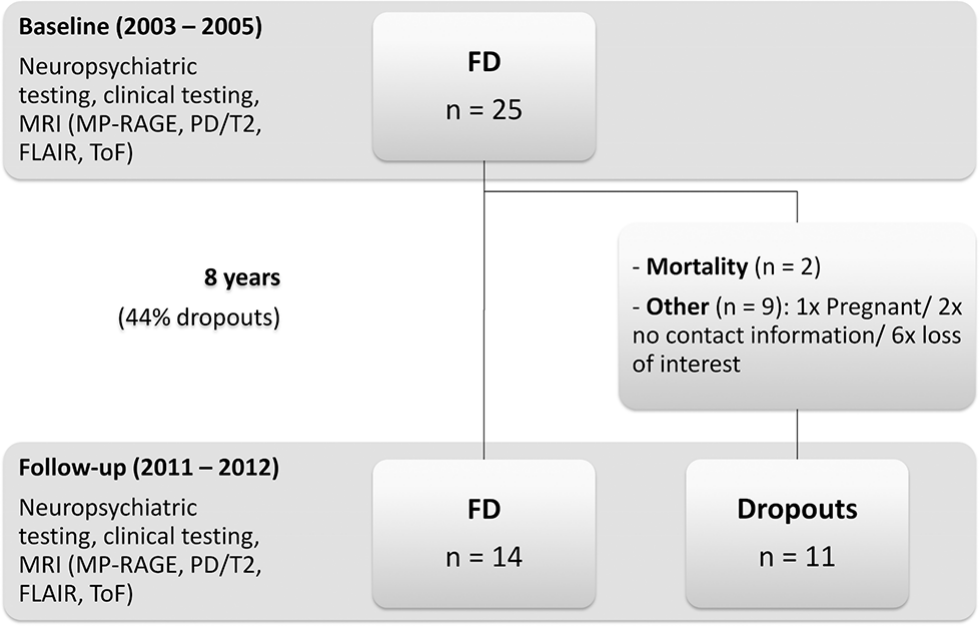
Study design of the longitudinal assessment. FD = Fabry disease; MRI = Magnetic resonance imaging.

This longitudinal cohort study was approved by the local ethics committee of the Landesärztekammer Rheinland-Pfalz in Mainz and all patients gave their written informed consent. Participants were enrolled at the Children’s Hospital, University Medical Center of Mainz. Baseline assessment was performed from 2003–2005 and follow-up assessment took place 8 years after baseline assessment from 2011–2012 (Fig 1). At baseline, 25 clinically affected classical FD patients (10M, age 37.9 years ± 10.8) were included in the study [5]. Patients had on average moderate disease severity [11], and standard recommended methods were used for enzymatic and molecular diagnosis of FD [12]. At follow-up, 14 patients (4M, age 46.1 ± 10.8 years) participated from the initial study group (Table 1). All patients at follow-up were classical FD patients with classic mutations; R301x, Q157x, A288D [13], R227x, W340x [14], c.945del21, A35OP, Q321x [15], IVS2+1G>A [16], R220x [17], and W236c [18]. At follow-up patients had, on average, moderate disease severity. 3 cerebrovascular events had occurred (stroke/transient ischemic attack), 8 patients had renal dysfunction (amount of patients with renal insufficiency, proteinuria and/or dysfunctional creatinine clearance as defined by the glomerular filtration rate according to Cockroft-Gault), and 8 patients had cardiac dysfunction (cardiomyopathy/arrhythmias). Cerebrovascular events and cardiac dysfunction did not increase from baseline to follow-up and renal dysfunction as determined by the globular filtration rate according to Cockroft-Gault improved from 71% to 57% of the patients [19], which can be explained by the enzyme replacement therapy (ERT) that most of the patients received from baseline to follow-up. Of 14 patients included in the follow-up assessment, 10 received ERT as a treatment for the symptoms of FD throughout the whole assessment, 2 patients received antidepressants at baseline, and an additional 2 patients received antidepressants at follow-up.

**Table 1:** Group comparisons for the neuropsychiatric parameters between baseline and follow-up.

	Baseline	Follow-up
N	.	14 (4M)
**Age at baseline (years)**	39 (19–55)	47 (27–64)
**Education (years)**	12.5(8–20)	.
**Dementia screening**	30 (27–30)	29.5 (24–30)
**Depression (#)**	7 (50%)	3 (21.4%)
Mild	6	2
Moderate	1	1
**Depression severity**	7.5 (0–27)	3 (0–21)
**Memory**		
Learning	62 (29–67)	58 (33–73)
Long term memory		
- Free recall	13.5 (3–15)	13.5 (6–15)
- Recognition	15 (12–15)	15 (13–15)
**Visual memory**		
Visual learning	37 (33–41)	36 (18–41)
Long term visual memory	35 (36–41)	31.5 (16–40)
**Psychomotor performance & attention**	19.7 (13.6–54)	22.5 (12–37)
**Executive functions**	46.4 (34–89)	56 (34–99)

Values are medians and ranges; group comparisons are controlled for gender; Dementia screening = Mini mental state examination; Depression = Hamilton depression rating scale-17; Memory = Auditory verbal learning test (Learning = immediate recall; Long term memory = delayed recall and recognition memory); Visual memory = Wechsler memory scale- Revised (Visual learning = immediate recall; Long term visual memory = delayed recall); Psychomotor performance & attention = Trail making test-A; Executive functions = Trail making test—B.

Reasons for patient dropout included: pregnancy, mortality, lack of contact information, and loss of interest (Fig 1). Both deceased patients had moderate disease severity and were not significantly more affected than other study patients [9]. Both deceased patients were middle-aged men who were on ERT. They had moderate disease severity and were not significantly more affected by cardiac dysfunction or cerebrovascular events than other study patients [9]. They were, however more affected by renal insufficiency than other dropouts or patients included at follow-up. Overall, non-deceased dropouts were not more severely affected than mean disease severity of patients included at follow-up. Non-deceased dropouts did not suffer more frequently from cerebrovascular, renal, or cardiovascular dysfunction, and also did not suffer more often from depression (after controlling for gender).

### Neuropsychological and psychiatric assessment

To assess learning and long term (free recall and recognition) memory we used the German version of the Rey Auditory Verbal Learning Task (AVLT [20]) in form of number of items remembered or recognized correctly. The German version of the Wechsler Memory Scale Revised (WMS-R) subtest visual reproduction was used to assess visual memory as raw scores of items correctly remembered immediately after presentation of the items (visual learning) and after a delay (long term visual memory [21]). The reaction times on the Trail Making Test A and B (TMT-A and-B) were used as measures for attention, psychomotor performance, and executive functions [22]. The mini mental state examination (MMSE) is a dementia screening tool and was used to gauge cognitive function [23]. To assess depression severity the Hamilton rating scale for depression was used (HAMD-17 [24]). Cut-offs for depression severity were defined as generally recommended [25]. Normal ranges for neuropsychiatric testing differ between age groups, gender, and/or education. Therefore normal ranges for AVLT, WMS-R visual reproduction, and TMT as well as cut-off scores for MMSE and HAMD-17 are provided in S1 Table.

### MRI data acquisition

Baseline, as well as follow-up data was obtained from a 1.5 T Magnetom Sonata system (Siemens, Erlangen, Germany). Standard 3D T1 Magnetization Prepared Rapid Gradient Echo (MP-RAGE)-weighted sequence (TR/TE 1900ms/16ms, matrix 512 x 512) was used for hippocampal volume (HV) analysis, FLAIR-weighted (TR/TE 9000ms/108ms, slice thickness 6 mm, matrix 512 x 448) sequence was performed for determination of white matter lesions (WMLs), magnetic resonance angiography (MRA) time-of-flight (ToF)-sequence (TR/TE: 40ms/4.97ms, slice thickness 0.8 mm, matrix 512 x 384) was assessed for means of measuring arterial diameters, and PD/T2 sequence (TR/TE 1/TE 2: 4500ms/15ms/100ms, matrix 256 x 256) to exclude further brain abnormalities.

### Hippocampus volumetry

For hippocampus measurement Analyze® Software (Version 8.1; Biomedical Imaging Software System, Mayo Foundation for medical education and research, Rochester) was used. Hippocampi were manually traced slice-by-slice on the default coronal view of MP-RAGE sequences for each hemisphere according to the Pruessner standardized protocol [26]. An experienced rater (I.L.) traced HVs for both baseline and follow-up. The rater was blinded to the time of measurement by randomly assigning numbers to the baseline and follow-up scans, which were analyzed consecutively (as in subsequently described WML- and artery determination). HV and WMLs (described below) were adjusted to total brain size by use of the well-established automated Brain Extraction Tool (BET) on T2 sequences [27] implemented in FSL (FMRIB Software Library v5.0 [28]). HV and WMLs are presented as the relative ratio of manually traced HVs, or WMLs divided by the brain volume in cm^3^ as obtained from BET.

### White matter lesions

WMLs were determined on the transversal FLAIR-sequences using the Analyze® 8.1 Software. WML boundaries were manually traced slice-by-slice by an experienced rater (A.B.) and were defined as bright lesions (>2mm) of the white matter or basal ganglia. Slice volumes were summed (ml) for every participant, and the relative ratio with BET was calculated (as previously described).

### Artery diameter assessment

Diameters of the larger cerebral arteries were measured manually by an experienced rater (I.L) on the sagittal ToF sequence using the Sectra Workstation IDS7 (Version 16.1.2.1103; Linköping, Sweden). Diameters were measured perpendicular to the vessel [29]. The following arteries were defined: For the basilar artery, the average of caudal (just above confluence of the vertebrate arteries), medial (middle of basilar artery), and rostral (just before bifurcation) diameter was calculated; the left and right posterior cerebral artery diameters were determined in the middle of P2 segment; the left and right internal carotid artery diameters were measured in C7 segment, 5 mm before bifurcation into middle and anterior cerebral artery; the left and right middle cerebral artery diameters were measured in the middle of M1 segment; and finally, the left and right anterior cerebral artery diameters in the middle of A1 segment. In some cases arteries were not assessable as defined in Table 2.

**Table 2:** Descriptive data and group comparisons of the MR-imaging parameters between baseline and follow-up.

		Baseline	Follow-up
**Hippocampal volume**	R	1363 (1272–1785)	1264 (1108–1456)**
	L	1376 (1255–1731)	1252 (1086–1440)**
**White matter lesions**		125 (0–1636)	1026 (0–2781)
**Cerebral arteries:**			
Basilar		3.1 (1.6–4.4)ᵃ	3.1 (2.2–4.5)
Posterior	R	2 (1.2–3.2)ᵃ	2 (0.8–2.8)
	L	2 (0.8–2.6)ᵃ	2.2 (0.8–2.6)
Carotid	R	2.6 (1.6–3.6)ᵃ	2.7 (2–3.6)
	L	2.6 (1.8–3.4)ᵃ	2.9 (1.2–3.4)
Middle	R	2.4 (1.2–2.8)ᵃ	2.4 (2.2.–2.8)
	L	2.2 (1.6–2.8)ᵃ	2.2 (2–2.6)
Anterior	R	1.8 (0.6–2.4)ᵃ	1.8 (1.2–2.6)ᵃ
	L	1.8 (1–2.6)ᵃ	1.8 (1.2–2.4)

Values represent medians and ranges in mm^3^ (hippocampal volume and white matter lesions) and mm diameter (arteries); group comparisons are controlled for gender; R = Right; L = Left.

**significant at a <.01 level.

ᵃ n = 12.

### Statistics

For statistical analysis we used IBM SPSS statistics 22.0 software (Ehningen, Germany). All statistical analyses were performed with gender as a covariate, except otherwise specified, as it has been found to have significant influence on FD development [3]. Analyses were done with the 14 participants that have both baseline and follow-up assessment. We performed repeated measures analysis of covariance, Spearman’s rank correlation coefficient, and partial correlation coefficients [3]. In an exploratory analysis we computed non-parametric Mann-Whitney-U tests and robust regression analyses to control for outliers.

## Results

### Neuropsychiatric symptoms

Descriptive data and group comparisons of the neuropsychiatric parameters for baseline and follow-up are described in Table 1. We did not find significant differences between baseline and follow-up performance for any cognitive task, and we did not find differences between baseline and follow-up on the depression scale (HAMD-17), after controlling for gender. Although we did not find statistically significant differences between baseline and follow-up on depression severity or frequency, clinically 50% of the 14 FD patients enrolled at both assessments showed clinically relevant depressive symptoms at baseline (defined as HAMD-17 > 7); only 21.4% of the FD patients still showed depressive symptoms at follow-up (Table 1). Of the 7 patients having depression at baseline 3 received antidepressants and had no symptoms of depression at follow-up. Patients with or without depression, or with changes in depression severity over time, did not differ in age. Depression neither correlated with neuropsychological measures at baseline, nor at follow-up except with long term memory at follow-up, after controlling for gender (r = .62, p = .024). Robust regression analyses with depression (at follow-up) and gender as predictors and long term memory (at follow-up) as an outcome variable were not significant.

### Brain structural alterations

**Fig 2:**
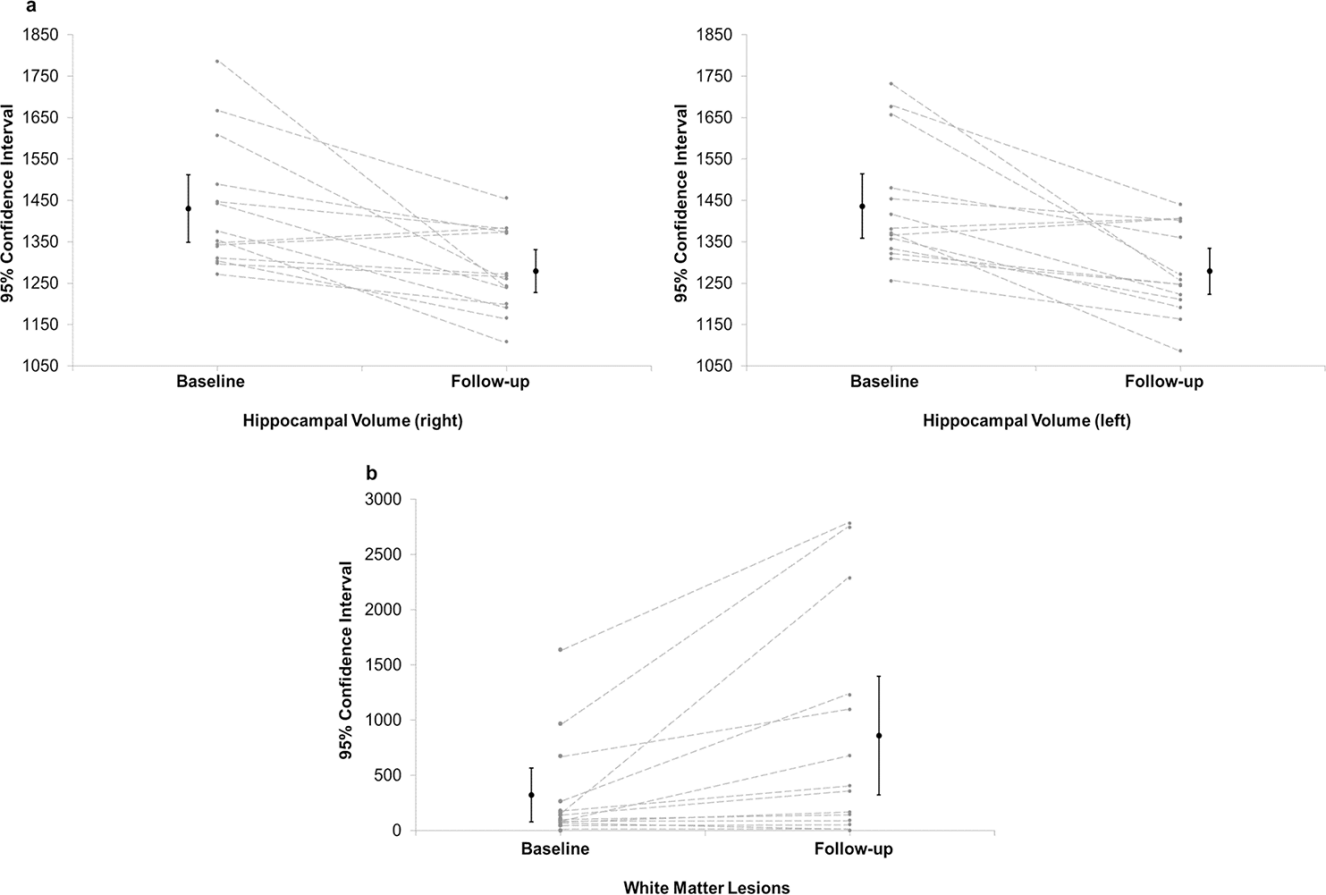
Hippocampal volume and white matter lesions at baseline and follow-up. 2a) Hippocampal volume 95% confidence intervals (CI): Baseline (right): 1349–1512 mm^3^, follow-up (right): 1227–1331 mm^3^; baseline (left): 1358–1514 mm^3^, follow-up (left): 1223–1335 mm^3^; 2b) White matter lesions 95% CI: Baseline 79–566 mm^3^, follow-up 322–1396 mm^3^.

The following results have been controlled for gender, unless stated otherwise. Table 2 shows descriptive data of the imaging parameters. There is a significant decline in HV from baseline to follow up in both right and left HV (f(1,12) = 13, p = .004 and f(1,12) = 14.1, p = .003, respectively; Fig 2A). Baseline and follow-up WMLs were significantly associated with older age (baseline: r = .57, p = .043; follow-up: r = .612, p = .026). Also, baseline WMLs were highly significantly correlated with follow-up WMLs (r = .82, p = .001). However, even though WMLs showed a significant difference between baseline and follow-up before controlling for gender (t(13) = -2.8, p = .014, Fig 2B), it failed to reach significance after controlling for gender (f(1,12) = 3.1, p = .106). Right and left HV at baseline and follow-up were not associated with age. Robust regression analysis with the larger intracranial arteries (difference between baseline and follow-up) and gender as predictors and WML as outcome variables showed no significance, except between left anterior cerebral artery and WMLs (t(9) = 3.1, p = .013). Robust regression analyses with HV as outcome variables showed no significance. There was no significant interaction effect or correlation between increased WML load and HV atrophy from baseline to follow-up. None of the cerebral artery diameters measured changed significantly from baseline to follow-up. Controlling for cerebrovascular events did not change the results.

### Association between brain structural, neuropsychiatric, and descriptive parameters

Spearman correlations showed significant associations between HV (left and right) difference (baseline minus follow-up) and difference of recognition, as measured with the memory test AVLT (right: r = .595, *p* = .025; left: r = .683, *p* = .007). Furthermore, WML difference correlates with difference in performance on TMT–B (r = .534, *p* = .049). Partial correlations after controlling for gender showed no significant correlations, but showed tendencies (r >. 4) towards the previously-described associations before controlling for gender. Brain structural parameters showed no association with depression severity or frequency, with occurrence of pain as measured with the brief pain inventory, with renal involvement (creatinine clearance), with cerebrovascular events or cardiovascular disease (cardiomyopathy and/or arrhythmias) [30].

## Discussion

Our analyses revealed no differences in cognitive performance between baseline and follow-up. In a previous publication, there were no clinically relevant cognitive deficits apparent at baseline, compared to controls [9]. Slight impairment in attention and executive functions were evident, but after correction for depression severity only mild attentional deficits were significant. Furthermore Bolsover and colleagues (2014) concluded in a recent review that in FD, only mild cognitive deficits were notable [8]. Therefore, cognitive decline could be surprisingly limited in middle aged FD patients. Interestingly, despite the limited cognitive decline, we found highly significant hippocampal atrophy of 11% over the 8 years of our longitudinal assessment; this is consistent with our previous findings [5] and with post-mortem case-studies in FD, (i.e. [31, 32]. In healthy adults HV development has been described as an inverted-U-relationship, first increasing in young adulthood, plateauing in middle age, and with accelerated HV atrophy from 60 years onwards with an atrophy rate of around 1% per year [7, 33]. Consequently hippocampal atrophy as seen in our investigation (11%) typically would not be expected in middle aged adults between 27 to 64 years of age (median 47 years) at follow-up. Remarkably, 11% atrophy even exceeds normal atrophy rates of older individuals of 60 years and older. Autopsy studies in FD patients have repeatedly shown severe globotriosylceramide (Gb3) accumulation in the neurons and ganglion cells of the hippocampus, which could ultimately lead to functional deterioration of the cells, i.e.[31, 34], and might even lead to compromised energy metabolism, oxidative stress and cellular death. Gb3 deposition in FD is the result of the inherited reduction of α-Galactosidase and causes clinical manifestation in early childhood, with a slight delay in girls [2]. Early accumulation of Gb3 in the lysosomes of cells and subsequent cellular death could thus be responsible for such early hippocampal atrophy as observed in our study. Neuropathic pain and major depression have also been shown to be strongly related to reduced HV in otherwise healthy subjects [35]. Given that pain and depression are frequent symptoms in FD, the impact of both on HV decline seems expected. However, neither pain, nor depression was associated with hippocampal decline in our cohort. Hippocampal atrophy in Alzheimer’s disease, a disease well-known for its pronounced HV decline, is associated with marked cognitive decline, especially in episodic memory. Even in healthy aging, normal degrees of cognitive decline in episodic memory, attention, executive functions, and psychomotor performance, as well as the involvement of differential brain areas is expected [36]. However, HV decline in our investigation was not associated with any neuropsychiatric parameters. Other FD relevant factors that have previously been shown to be associated with hippocampal atrophy such as cardiovascular disease or the occurrence of cerebrovascular events were also not associated with HV decline [37]. Therefore, we conclude that HV decline in FD might not be age-related and might be functionally compensated in our FD cohort. HV decline in middle aged FD patients might predict consecutive cognitive decline. Literature further postulates that hippocampal atrophy might be a result of increased WML load in FD [38]. However, we did not find associations of HV and WML-load in our study. We demonstrated that WML increases were associated with older age and higher WML-load at baseline, but this did not reach statistical significance when baseline was compared to follow-up. An increase of WML in older age has been hypothesized to be involved in developing late-life depression, but a recent review and meta-analysis found that the effect is most likely small [10]. Depression in our middle aged FD cohort was not associated with cognitive performance, or with any brain structural parameters measured. Of note, even though not statistically significant: 50% of the FD patients showed clinically relevant depressive symptoms at baseline, but only 21% had depression at follow-up. This finding can be explained by the fact that depressive patients received symptomatic antidepressive treatment after enrollment. Depressive symptoms naturally fluctuate over the course of time, which might have caused the non-significant difference between baseline and follow-up. Nonetheless, future studies should address depression in Fabry disease, as it can be a major burden on patients and their families, as well as the patient’s suffering due to a painful disease, which ultimately can significantly affect quality of life [39]. Placebo controlled studies are needed to address antidepressant treatment options in patients with Fabry disease.

Recent literature has shown that dolichoectasia of the larger intracranial arteries, especially the basilar artery, can be the earliest marker of cerebrovascular involvement and might therefore be a potential screening tool in FD [3, 4]. However, in a secondary analysis we found that although baseline arteries were dilated in our FD patient cohort compared to controls [3], longitudinal results show no statistically significant increase in arterial diameters over 8 years. Still, our results show that greater diameters of the anterior cerebral artery significantly predicts WML load. Existing literature is still unclear about whether cerebrovascular disease predicts white matter disease, or if both conditions are a comorbid presentation in non-Fabry cohorts [40]. Furthermore, in Fabry-studies results have shown no or incomplete associations between WML and cerebral artery diameters [4]. Therefore, further investigations addressing the relationship of WML and cerebral arteries in FD are needed.

A limitation to our findings is the significant dropout rate of 44%, which decreased our FD cohort to 14 participants. The majority of the dropouts can be explained by the emergence of new FD centers in several locations throughout Germany during our follow-up interval of 8 years. Because we assessed patients from all over Germany at baseline, motivation for travelling to a more distant Fabry center at follow-up was most likely low. Mortality of the 25 patients included at baseline only accounted for 4% of the dropouts and these patients were not more severely affected than the mean severity at baseline, which suggests no FD severity bias in our results. Considering that our sample was rather small (n = 14), results might be susceptible to type II errors. Although we analyzed the relationship between hippocampal atrophy and several factors known to possibly alter HV (i.e. pain, depression, cardiovascular disease, cerebrovascular events, WML, artery diameter), we cannot rule out that other factors such as diabetes, obesity, obstructive sleep apnea, vitamin b12 deficiency etc. might have influenced the results [30]. However, as these factors are not related to FD, discussing all of them would be beyond the scope of this text.

This investigation demonstrates clinical stability in cognitive function, while pronounced hippocampal atrophy is apparent throughout the 8 years. Our middle-aged FD patients seem to compensate successfully for progressive HV loss. However, since hippocampal atrophy was 11% over eight years, we expect FD patients to show further hippocampal atrophy, eventually passing a threshold of cognitive decline much earlier than the healthy population. Notably, marked hippocampal atrophy clearly exceeding age-associated volume decline provided further evidence of regional neuronal involvement in FD. The heterogeneous WML increases were associated with older age and higher WML-load at baseline, but not with HV, suggesting that WML involvement and HV decline are independent processes occurring in FD.

## Supporting Information

S1 Table Normal ranges of neuropsychiatric testing. (DOCX)
