# Supplementary material for: Eight-Year Follow-Up of Neuropsychiatric Symptoms and Brain Structural Changes in Fabry Disease
Source: PLoS One. 2015 Sep 4;10(9):e0137603. doi: 10.1371/journal.pone.0137603 (PMC4560446; doi:10.1371/journal.pone.0137603)
Supplement: S1 Table — (DOCX) [file pone.0137603.s001.docx]

| **AVLT** | Age ranges | 20-29 | | 30-39 | | | 40-49 | | 50-59 | | | 60-69 | |
| --- | --- | --- | --- | --- | --- | --- | --- | --- | --- | --- | --- | --- | --- |
|  | Gender | m | f | m | | f | m | f | m | f | | m | f |
|  | Immediate recall | 54.9 ±7 | 55.3±6.6 | 46±10.9 | | 55.9±6.3 | 47.5 ±8.3 | 52.1±7.1 | 47.6 ±8.5 | 47.6±7.7 | | 36.7±8.4 | 49±7.1 |
|  | Delayed recall | 10.6 ±2.4 | 11±2 | 10.4±2.3 | | 12.2±2.5 | 10.5±2.7 | 11.1±2.3 | 10±2.6 | 10.2±2.7 | | 7.1±3.8 | 10.3±2.3 |
|  | Recognition | 14.2±0.8 | 14.4±0.8 | 13.5±1.5 | | 14.2±1.7 | 14.2±1 | 14.4±0.8 | 13.9±0.9 | 13.7±1.1 | | 12.4±2.8 | 13.8±1.1 |
| **WMS-R** | Age ranges | 20-25 | | 26-34 | | | 35-44 | | 45-54 | | | 55-64 | |
|  | Vis. Reproduction I | 36.7±3 | | 36.4±3.8 | | | 36.1±3 | | 35.4±5 | | | 31.2±6.2 | |
|  | Vis. Reproduction II | 35.1±3.1 | | 34.6±5.2 | | | 32.9±5.3 | | 32.6±6.4 | | | 28.1±7.5 | |
| **TMT** | Age ranges | 25-34 | | 35-44 | | | 45-54 | | 55-59 | | | 60-64 | |
|  | Education | 0-12+ | | 0-12+ | | | 0-12+ | | 0-12 | 12+ | | 0-12 | 12+ |
|  | TMT-A | 24.4±8.7 | | 28.5±10.1 | | | 31.8±9.9 | | 35.1±10.9 | 31.7±10.1 | | 33.2±9.1 | 31.3±7 |
|  | TMT-B | 50.7±12.4 | | 58.5±16.4 | | | 63.8±14.4 | | 78.8±19.1 | 68.7±21 | | 74.6±19.6 | 64.6±18.6 |
| **MMSE** | Score | 27-30 | | | 21-26 | | | 11-20 | | | 0-10 | | |
|  | Cognitive Function | Normal | | | Mild impairment | | | Moderate impairment | | | Severe impairment | | |
| **HAMD-17** | Score | 0-7 | | | 8-16 | | | 17-23 | | | >23 | | |
|  | Depression severity | No Depression | | | Mild depression | | | Moderate depression | | | Severe depression | | |

**S1 Table. Normal ranges of neuropsychiatric testing**

Scores of AVLT, WMS-R, and TMT are presented as means ± standard deviation; Age ranges and education are presented in years; m = male, f = female

AVLT = Rey Auditory Verbal Learning Task; scores of AVLT are presented as numbers of items remembered or recognized correctly

WMS-R = Wechsler Memory Scale – Revised; scores of WMS-R are raw scores calculated from items of pictures remembered correctly

TMT = Trail Making Test; scores are reaction times in seconds

MMSE = Mini Mental State Examination

HAMD-17 = Hamilton rating scale for Depression
